# Serological Responses to *Streptococcus pyogenes* Vaccine Candidate Antigens Suggests That *Streptococcus dysgalactiae* Is the Predominant Cause of Lower Limb Cellulitis

**DOI:** 10.1093/ofid/ofae272

**Published:** 2024-06-11

**Authors:** Michael Taggart, Kristyn Langworthy, Siong Hui, Conchita Boyder, Alma Fulurija, Michael Morici, Edward Raby, Laurens Manning

**Affiliations:** Department of Infectious Diseases, Fiona Stanley Fremantle Hospitals Group, Murdoch Western Australia, Australia; Department of Infectious Diseases, Fiona Stanley Fremantle Hospitals Group, Murdoch Western Australia, Australia; Department of Infectious Diseases, Fiona Stanley Fremantle Hospitals Group, Murdoch Western Australia, Australia; Department of Microbiology, PathWest Laboratory Medicine, Fiona Stanley Hospital, Murdoch, Western Australia, Australia; Telethon Kids Institute, University of Western Australia, Perth, Western Australia, Australia; Wesfarmers Centre of Vaccines and Infectious Diseases, Telethon Kids Institute, University of Western Australia, Perth, Western Australia, Australia; Telethon Kids Institute, University of Western Australia, Perth, Western Australia, Australia; Wesfarmers Centre of Vaccines and Infectious Diseases, Telethon Kids Institute, University of Western Australia, Perth, Western Australia, Australia; Department of Infectious Diseases, Fiona Stanley Fremantle Hospitals Group, Murdoch Western Australia, Australia; Department of Microbiology, PathWest Laboratory Medicine, Fiona Stanley Hospital, Murdoch, Western Australia, Australia; Medical School, Faculty of Health and Medical Sciences, The University of Western Australia, Crawley, Western Australia, Australia; Department of Infectious Diseases, Fiona Stanley Fremantle Hospitals Group, Murdoch Western Australia, Australia; Telethon Kids Institute, University of Western Australia, Perth, Western Australia, Australia; Wesfarmers Centre of Vaccines and Infectious Diseases, Telethon Kids Institute, University of Western Australia, Perth, Western Australia, Australia; Medical School, Faculty of Health and Medical Sciences, The University of Western Australia, Crawley, Western Australia, Australia

**Keywords:** cellulitis, serology, streptococcus, vaccine, GAS

## Abstract

**Background:**

A future *Streptococcus pyogenes* (Strep A) vaccine will ideally prevent a significant burden of lower limb cellulitis; however, natural immune responses to proposed vaccine antigens following an episode of cellulitis remain uncharacterized.

**Methods:**

We enrolled 63 patients with cellulitis and 26 with invasive beta hemolytic streptococci infection, using a multiplexed assay to measure immunoglobulin G against Strep A vaccine candidate antigens, including: streptolysin O (SLO), deoxyribonuclease B (DNB), group A carbohydrate (GAC), C5a peptidase (ScpA), cell envelope proteinase (SpyCEP), and adhesion and division protein (SpyAD). Responses in the invasive cohort were used to predict the infecting etiology in the cellulitis cohort.

**Results:**

Of 41 patients with cellulitis and paired serological samples, 68.3% had evidence of beta hemolytic streptococci infection by conventional anti-SLO and/or anti-DNB criteria. A positive serological response to at least 1 of the tested antigens was seen in 78.0% of the cellulitis cohort. Individually, anti-SLO (58.5%), anti-SpyAD (46.3%), and anti-ScpA (39.0%) were the most common. Based on principal component analysis, increases in these 3 antibodies, without responses to DNB, GAC, and SpyCEP characterized *Streptococcus dysgalactiae subspecies equisimilis* (SDSE) infection.

**Conclusions:**

SDSE appears to be the predominant cause of lower limb cellulitis. Effective Strep A vaccines incorporating antigens that provide additional cross protection against SDSE may prevent a significant burden of lower limb cellulitis.

Cellulitis refers to an acute infection of the dermis and subcutaneous tissue manifesting as spreading erythema, swelling, warmth, and/or pain [1, 2]. The 2017 Global Burden of Disease project found that the death rate from cellulitis had rapidly increased over the preceding decade [3]. This has been paralleled by an increase in hospital presentations for first episodes of lower limb cellulitis in Australia [4]. Repeated infections are common with 1 longitudinal cohort study finding a cumulative 5-year incidence of first, second, and third recurrences of 13.9%, 35.9%, and 52.9% [5]. In this study, hospital stay increased from 3 days for primary episodes to 5 days for recurrences [5]. As such, strategies targeted at secondary prevention are likely to provide substantial benefit in terms of morbidity and mortality. Indeed, the use of penicillin and compression as secondary prophylaxis are effective approaches in reducing cellulitis recurrence [6, 7].

It is thought that *Streptococcus pyogenes* (Group A Streptococcus; Strep A), *Streptococcus dysgalactiae subspecies equisimilis* (SDSE), and *Streptococcus agalactiae* (Group B Streptococcus; GBS) account for a high proportion of cellulitis cases. Quantification of the proportion caused by these beta hemolytic streptococci (BHS) has been made through various sampling strategies. A systematic review of observational studies on bacteremia in patients with cellulitis and erysipelas found that 6.5% of blood cultures were positive [8]. Of those, non-Strep A BHS were the most common, accounting for 37% [8]. Strep A was identified in 6.5% only [8].

Antibodies against streptolysin O (SLO) and deoxyribonuclease B (DNB) are often used to provide evidence of recent Strep A infection. A rise in anti-SLO is typically seen 1 week after BHS infection, peaking at 3 to 5 weeks, and returning to baseline levels by 6 to 12 months [9, 10]. A rise in anti-DNB often occurs slightly later, at approximately 2 weeks, and peaking at 6 to 8 weeks [11]. Although anti-DNB is thought to be specific for Strep A, anti-SLO may be elevated following infection with other BHS [12]. When applied as serological evidence of streptococcal cellulitis, a positive result is typically defined by a 0.2log10 increase between acute and convalescent samples, in addition to a convalescent value exceeding 200 IU/mL, or ≥200 IU/mL in both samples [13, 14]. Using these criteria, a prospective study of inpatients with cellulitis in Finland found that 69% had evidence of BHS infection [13].

Supplementing serological responses with culture results only identifies a limited number of additional cases of BHS cellulitis. In another prospective study from Scandinavia that used tissue sampling, blood culture, and serology, BHS were found in 72% of patients with cellulitis [14], consistent with findings from earlier studies [12]. Among those with a swab taken from an ulcer, wound, abrasion, toe web, or skin lesion, Strep A was found in 15.7%, SDSE was found in 22%, GBS in 3.1%, and mixed SDSE/GBS infection was found in 3.1%, accounting for 43.9% of all cellulitis cases [14]. Among those with *Staphylococcus aureus* and/or Gram-negative bacteria cultured from superficial swabs, 60% had confirmed evidence of streptococcal infection by serology, blood culture, or sterile tissue sampling, highlighting the questionable clinical significance of non-BHS cultured from nonsterile sites [14]. An underlying streptococcal etiology for cellulitis is further supported by high clinical response rates to penicillin for treatment and for prophylaxis [13–15].

Incorporating novel serological targets could help differentiate between different streptococcal species and refine the estimated burden of streptococcal cellulitis. Group A carbohydrate (GAC) is a highly conserved component of the Strep A cell wall and can be used to identify organisms belonging to this Lancefield group [16]. GAC antibody responses have been shown to peak later and at lower levels, compared to antibodies against SLO [16] and studies in the pediatric population have shown that antibiotics do not affect the GAC antibody response [17]. Streptococcal C5a peptidase (ScpA) is a Strep A cell-surface protease that cleaves complement C5a, thereby inhibiting leukocyte chemotaxis during infection [18]. Similarly, *Streptococcus pyogenes* cell envelope proteinase (SpyCEP) functions to evade the innate immune response by cleaving interleukin-8, subsequently impairing neutrophil recruitment [19]. *Streptococcus pyogenes* adhesion and division protein (SpyAD) has been discovered more recently and has been shown to be important in cell division and adhesion [20]. In a study examining the utility of an 8-plex assay in the diagnosis of Strep A infection, adults with Strep A bacteremia and children with acute rheumatic fever were found to have statistically significantly elevated levels of immunoglobulin G (IgG) against ScpA, SpyCEP, SpyAD, GAC, SLO, and DNB compared to age-matched controls [21].

These additional serological targets have not yet been explored in cellulitis. In this study, we aimed to estimate the burden of lower limb cellulitis caused by BHS using a 6-plex assay measuring IgG against SLO, DNB, ScpA, SpyCEP, SpyAD, and GAC. We aimed to predict the likely infecting organism in each patient with cellulitis (BHS vs non-BHS, and specific BHS organism) by measuring these same serological targets in a group of patients with microbiologically confirmed invasive BHS infection (Strep A, SDSE, and GBS). Based on previous studies [12–14], we hypothesized that serological evidence of BHS infection would be present in more than two thirds of patients. We also hypothesized that SDSE might account for a higher proportion of cellulitis cases than Strep A.

## METHODS

### Study Site and Population

Patients aged 18 years and older presenting with acute lower limb cellulitis to 3 public hospitals in Perth, Western Australia, were screened for recruitment between June 2020 and August 2022. Patients were identified through screening of admission diagnoses entered into the digital medical record. Patients with microbiologically proven invasive BHS disease were identified by the laboratory and were recruited to improve predictions of the likely pathogen in the cellulitis patient cohort. Patients unable to provide informed consent and those unavailable for convalescent serology collected were excluded. Written informed consent was provided by all participants. The study received ethical approval from the South Metropolitan Health Service Health Research Ethics Committee (approval number RGS3180).

### Case Definitions

A pragmatic definition of lower limb cellulitis was used [6]. Cases were defined by new-onset lower limb local warmth, tenderness, or acute pain with unilateral or bilateral erythema, in conjunction with a temporal link between symptoms and the more affected leg [22]. All cases were reviewed by an experienced clinician working in the infectious diseases department to exclude alternative diagnoses (eg, acute Charcot foot, other lower limb dermatoses). Invasive BHS infection was defined as culture of BHS from a normally sterile body site [23].

### Clinical Characteristics

Data were collected regarding cellulitis participant demographics, antimicrobial treatment, risk factors, prior episodes of cellulitis, length of stay, and peak C-reactive protein. Risk factors were based on prior published data [4] and were defined as: diabetes, tinea pedis, venous insufficiency, peripheral vascular disease, lymphoedema, previous coronary artery bypass grafting, heart failure, renal failure, and liver failure.

### Serology

The earliest available sample taken as part of the participant's routine clinical care was stored at −80 °C for acute serological analysis. Participants were requested to have a convalescent sample taken 4 weeks later (±1 week). A multiplexed electrochemiluminescence assay developed using the Meso Scale Discovery (MSD) platform for the Australian Strep A Vaccine Initiative was used to measure IgG antibody responses. Purified GAC and purified recombinant SpyCEP, SpyAD, ScpA, DNB, and SLO were bound to spots on a carbon electrode surface within each well of a microtiter plate. Nonspecific binding on plates was blocked with Blocker A solution followed by washing with wash buffer and addition of a 50 µL serum sample from each participant at a minimum dilution of 1:100 000. Serially diluted intravenous Ig was used to generate a standard curve, and 3 quality control samples were tested in parallel. After 2 hours, plates were washed and 50 µL of 1 µg/mL anti-human IgG SULFO-TAG detection antibody was added. After a further 1 hour and washing with buffer, 150 µL/well of MSD GOLD Read Buffer B was added to each well. A charge-coupled device camera (MSD Sector S 600 instrument) was used to detect electrochemiluminescence-generated light.

**Figure 1.**
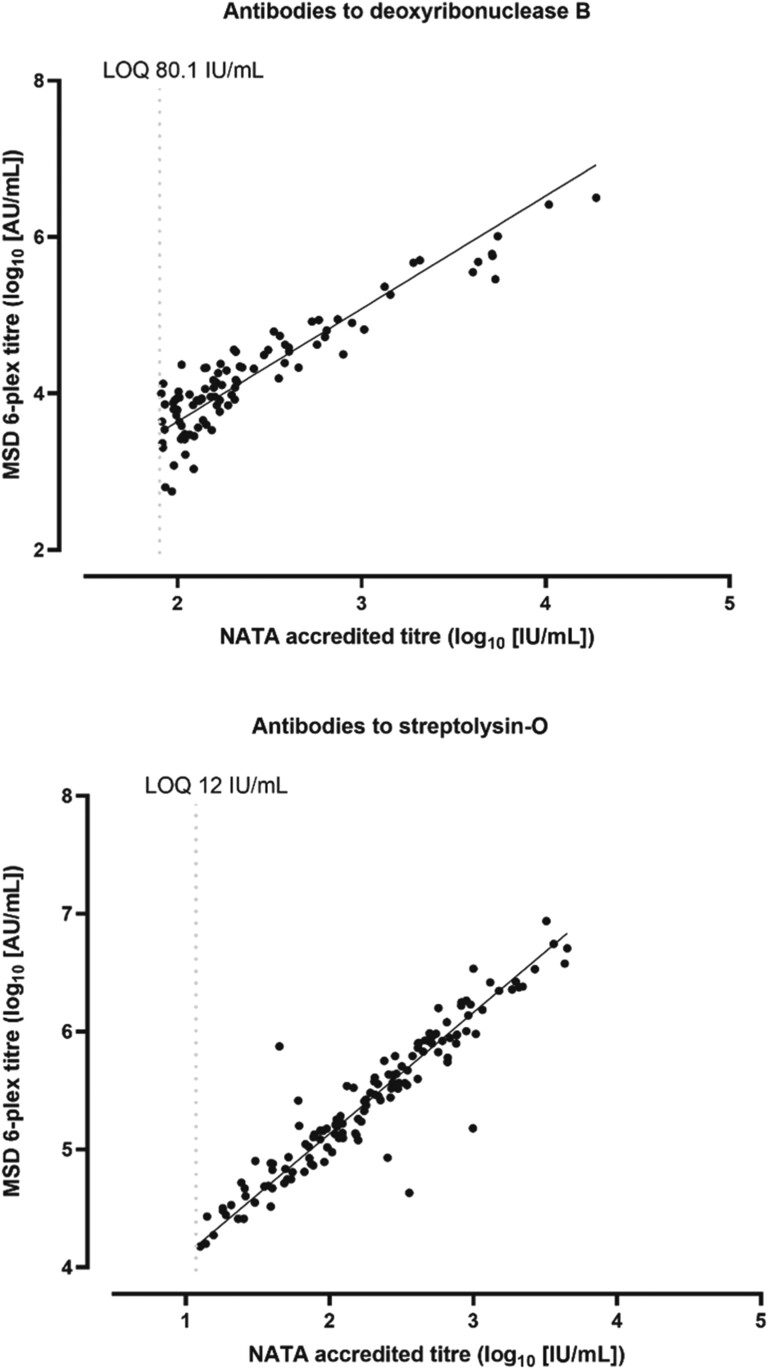
Deming regression analysis of log transformed anti-deoxyribonuclease B titer (top) and anti-streptolysin O titer (bottom) measured by multiplexed electrochemiluminescence in arbitrary units (MSD 6-plex; AU/mL) plotted against National Association of Testing Authorities measurement in international units (NATA; IU/mL). Abbreviation: LOQ, limit of quantification.

Quantification of the detected antibody was interpolated from a standard curve and reported in arbitrary units. MSD Discovery Workbench software (version 4) was used to process raw data. Four-parameter logistic regression curve fitting was used for the standard curves with 1/y^2^ weighting. Samples which produced results above the standard curve fit were reassayed at a higher dilution. The MSD platform output for the 6 antigens were provided as arbitrary units (AU/mL). To convert anti-SLO and and-DNB to standard international units (IU/mL), all samples were also assayed on a National Association of Testing Authorities accredited assay. The limit of quantification for this platform was 12 and 80.1 IU/mL for anti-SLO and anti-DNB, respectively. After Deming regression, cutoff points representing levels >200 IU/mL were applied to the raw data from the MSD platform for each anti-SLO and anti-DNB (Figure 1). Because there were no National Association of Testing Authorities–established assays for the remaining analytes, these were analyzed as AU/mL.

### Statistical Analysis

Demographics and clinical characteristics were summarized by median and interquartile range (IQR) for continuous numerical variables and percentages for categorical variables. A Mann-Whitney test was used to compare median values for continuous variables and chi-square testing for comparisons between dichotomous variables.

For GAC, ScpA, SpyCEP, and SpyAD, a positive serological response was defined as a 0.2log10 increase in IgG level between acute and convalescent samples. Based on prior studies [13, 14], a positive serological response for anti-DNB and anti-SLO was defined as:

1. Anti-DNB; >0.2log difference between acute/convalescent with a convalescent value >11 757 AU/mL; OR both acute and convalescent anti-DNB >11 757 AU/mL (corresponding to a cutoff of >200 IU/mL)
2. Anti-SLO; >0.2log difference between acute/convalescent with a convalescent value >276 541 AU/mL; OR both acute and convalescent anti-SLO >276 541 AU/mL (corresponding to a cutoff of >200 IU/mL)

Serological responses were further characterized using unsupervised and supervised multivariate methods in R (package *ropls* v1.32.0). Briefly, each individual was categorized as “cellulitis,” “invasive Strep A,” “invasive SDSE,” or “invasive GBS.” For each of the 6 assays, a value representing the log10 difference between acute and convalescent values was centered and scaled by subtracting the mean and dividing by the standard deviation. Principal component analysis was performed to describe and visualize the whole dataset [24].

## RESULTS

### Clinical Characteristics

Sixty-three patients were recruited to the cellulitis arm and had baseline serology. Median length of stay was 6 days (IQR 3–10). Baseline characteristics, according to whether the presenting episode was the primary or recurrent episode of cellulitis, are shown (Table 1). Median age at enrollment was 65 years in both groups. Except for venous insufficiency, which was more frequent in recurrent cellulitis (11/37 vs 2/26; *P* = .04), there were no significant differences in risk factors between those presenting with recurrent infections compared with primary episodes. At baseline, the levels for anti-GAC and anti-SLO were both significantly higher in participants with recurrent infections (4.23 vs 3.80 log10 AU/mL, *P* = .025 and 5.44 vs 5.12 log10 AU/mL, *P* = .05). Nearly all (98.4%) participants received at least 1 beta lactam antibiotic at enrollment. A first-generation cephalosporin was the most common (45.2%). A second, non–beta lactam agent was used in 33.3% of participants. The most common non–beta lactam agent used was clindamycin.

**Table 1.** Clinical, Laboratory, and Serological Characteristics at Baseline of All Adults With Lower Limb Cellulitis According to Whether Presenting Episode Was a Primary or Recurrent Episode

Characteristic	Primary Episode (n = 26)	Recurrent Cellulitis (n = 37)	*P* Value
Age (y), median (IQR)	65 (53–76)	65 (53–76)	.79
Sex, male (n; [%])	17 (65.38)	26 (70.27)	.37
Risk factors			
Diabetes (n; [%])	9 (34.62)	14 (37.84)	.79
Tinea pedis (n; [%])	7 (26.92	17 (45.95)	.14
Congestive cardiac failure (n; [%])	5 (19.23%)	11 (29.73)	.37
Venous insufficiency (n; [%])	2 (7.69)	11 (29.73)	.04
Chronic liver disease (n; [%])	3 (11.54)	3 (8.11)	.65
Chronic kidney disease (n; [%])	6 (23.08)	3 (8.11)	.09
Lymphoedema (n; [%])	1 (3.85)	6 (16.22)	.13
Peripheral vascular disease (n; [%])	1 (3.85)	6 (16.22)	.13
Coronary artery bypass graft (n; [%])	1 (3.85)	4 (10.81)	.33
Laboratory data			
Peak C-reactive protein (mg/L), median (IQR)	161 (97.75–292.25)	187 (101–299)	.75
Invasive disease, n (%)	4 (15.38)	5 (13.51)	.34
Baseline serology results			
Anti-DNB, log10 AU/mL; median (IQR)	3.64 (3.44–4.19)	3.93 (3.45–4.33)	.13
Anti-DNB >200 IU/mL; n (%)	7 (26.92)	14 (37.84)	.37
Anti-GAC, log10 AU; median (IQR)	3.80 (3.66–4.08)	4.23 (3.81–4.57)	.025
Anti-ScpA, log10 AU; median (IQR)	4.57 (4.22–4.96)	4.81 (4.23–4.98)	.09
Anti-SLO, log10 AU; median (IQR)	5.12 (4.69–5.57)	5.44 (4.75–5.60)	.05
Anti-SLO >200 IU/mL; n (%)	8 (30.77)	18 (48.65)	.16
Anti-SpyAD, log10 AU; median (IQR)	4.61 (4.06–4.98)	4.81 (4.14–5.00)	.06
Anti-SpyCEP, log10 AU; median (IQR)	4.10 (3.81–4.62)	4.29 (3.81–4.63)	.17

Abbreviations: AU, arbitrary units; DNB, deoxyribonuclease B; GAC, Group A carbohydrate; IQR, interquartile range; ScpA, Streptococcal C5a peptidase; SLO, streptolysin O; SpyAD, *Streptococcus pyogenes* adhesion and division protein; SpyCEP, *Streptococcus pyogenes* cell envelope proteinase.

**Figure 2.**
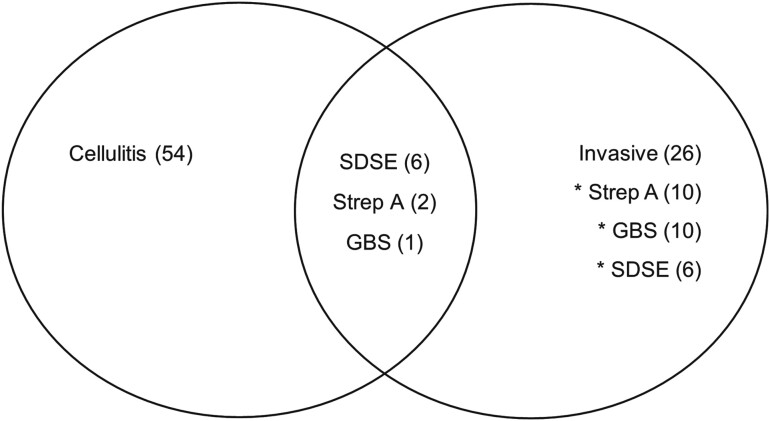
Venn diagram outlining the case definitions met by the recruited cohort of patients, including cellulitis without invasive disease (54), invasive disease without cellulitis (26), and cellulitis with invasive disease (9). Note that 1 patient met the criterion for invasive disease resulting from Strep A culture from a deep surgical tissue sample in the absence of bacteremia. Abbreviations: GBS, *Streptococcus agalactiae*; SDSE, *Streptococcus dysgalactiae subspecies equisimilis*; Strep A, *Streptococcus pyogenes*.

There were 35 participants with microbiologically proven invasive BHS infection and paired serological samples including 12 with Strep A, 12 with SDSE, and 11 with GBS. Blood cultures were drawn in 45 (71.4%) participants in the cellulitis cohort. Eight of these participants had confirmed bacteremia (17.8%), including 6 with SDSE, 1 with Strep A, and 1 with GBS (Figure 2). One patient met criteria for both invasive disease and cellulitis on the basis of Strep A cultured from deep tissue samples. Thirty participants had superficial swabs taken. SDSE was isolated from 3 participants (10.0%), GBS was isolated from 3 participants (10.0%), Strep A was isolated from 2 participants (6.7%), and Strep A/GBS co-infection in 1 participant (3.3%).

### Serology

**Figure 3.**
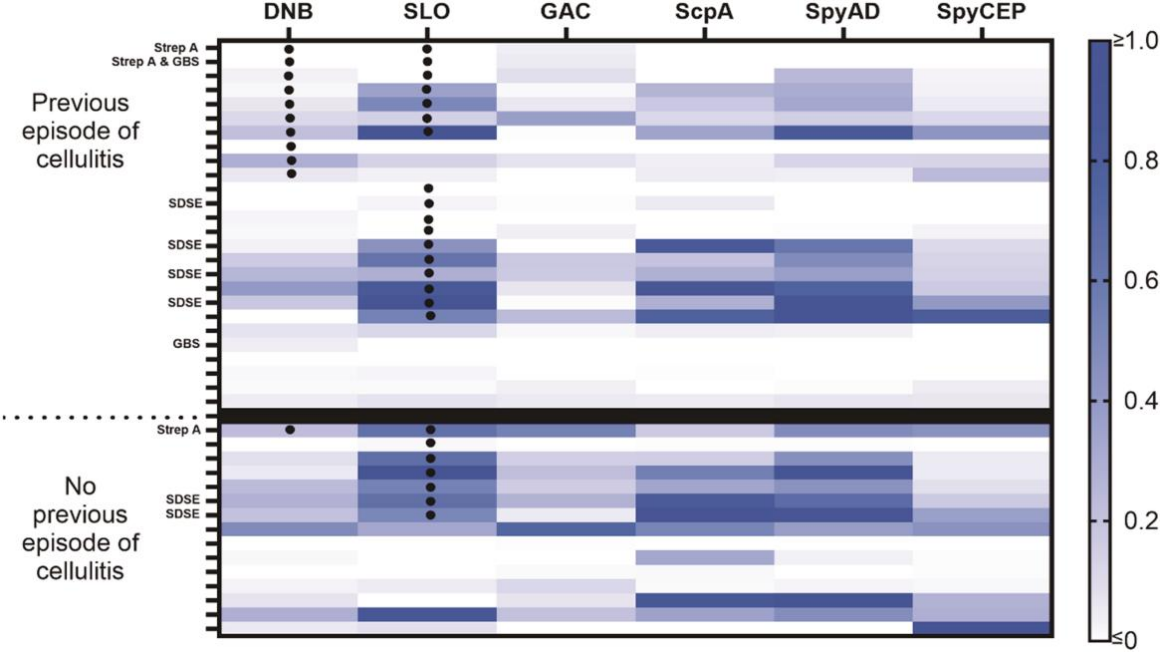
Heat map outlining the magnitude of antibody responses for each individual participant (denoted by ticks on vertical axis) separated by first or recurrent episode of cellulitis. Antibody responses are expressed as the logarithmic difference between acute and convalescent antibody levels. Dots represent a positive antibody response to streptolysin O or deoxyribonuclease B, accounting for participants who may not have had a significant change in levels between acute and convalescent samples, but where both were >200 IU/mL. The recovery of *Streptococcus pyogenes* (Group A Streptococcus; Strep A), *Streptococcus dysgalactiae subspecies equisimilis* (SDSE), and *Streptococcus agalactiae* (Group B Streptococcus; GBS) from blood cultures or swab is shown on the vertical axis. Abbreviations: DNB, deoxyribonuclease B; GAC, Group A carbohydrate; ScpA, Streptococcal C5a peptidase; SLO, streptolysin O; SpyAD, *Streptococcus pyogenes* adhesion and division protein; SpyCEP, *Streptococcus pyogenes* cell envelope proteinase.

Paired acute and convalescent serology was available in 41 participants with cellulitis. The median number of days between samples was 28 (IQR 27–31). A heat map outlining responses to each individual antigen among the cellulitis cohort is shown (Figure 3). A total of 32 participants (78.0%) demonstrated a positive serological response to at least 1 of the tested antigens, including 24 for SLO (58.5%), 19 for SpyAD (46.3%), 16 for ScpA (39.0%), 11 for DNB (26.8%), 10 for SpyCEP (24.4%), and 7 for GAC (17.1%). Of the 28 (68.3%) who fulfilled conventional anti-SLO and/or anti-DNB criteria, 17 met the criteria based on anti-SLO alone (41.5%), 4 on anti-DNB alone (9.8%), and 7 with both anti-SLO and anti-DNB (17.1%). There was no difference in the proportion of individuals lacking a serological response to any of the tested antigens according to primary or recurrent episodes (20.0% vs 23.1%, *P* = .87).

**Figure 4.**
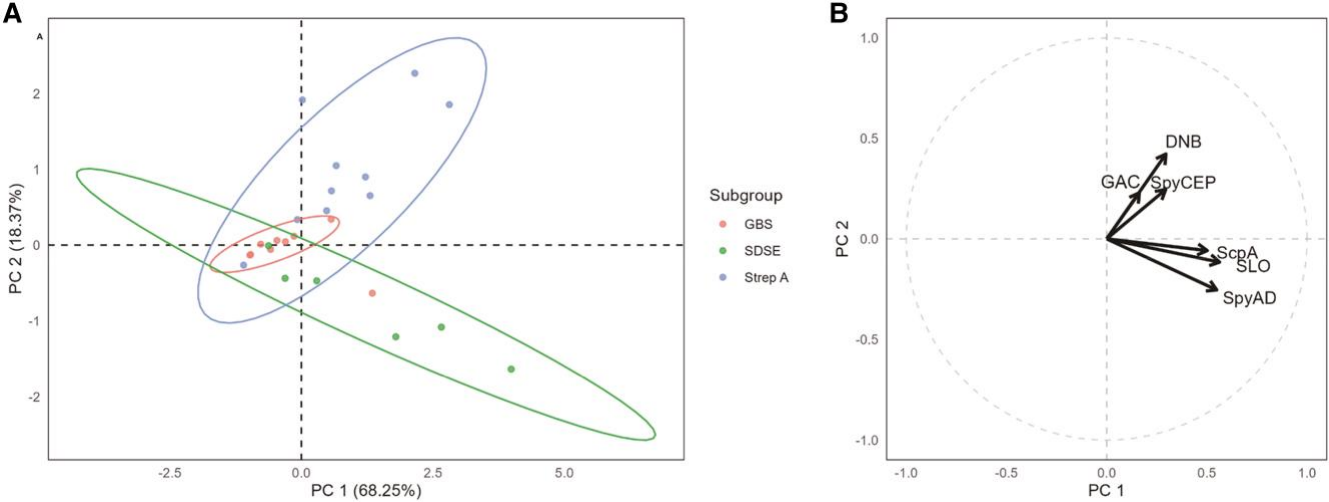
Principal component analysis. (*A*) Plot of individual scores for the first 2 components (PC1 and PC2). Individuals with invasive GBS (*Streptococcus agalactiae*, red), invasive SDSE (*Streptococcus dysgalactiae subspecies equisimilis,* green), and invasive Strep A (*Streptococcus pyogenes*, blue) are shown. (*B*) Loadings demonstrating the contribution of each antibody to principal component 1 (PC1) and principal component 2 (PC2). Abbreviations: DNB, anti-deoxyribonuclease B; GAC, anti-Group A carbohydrate; ScpA, anti-streptococcal C5a peptidase; SLO, anti-streptolysin O; SpyAD, anti-*Streptococcus pyogenes* adhesion and division protein; SpyCEP, anti-*Streptococcus pyogenes* cell envelope proteinase.

Three patients with invasive GBS showed no serological response to any of the tested antigens. Principal component analysis showed minimal serological responses in the invasive GBS group that was then used as a control in secondary analyses (Figure 4). There was a range of responses in invasive Strep A and SDSE clusters with clear separation of distinctive serological markers. Antibodies to GAC, DNB, and SpyCEP were positively associated with both principal component 1 (PC1) and principal component 2 (PC2). Those with invasive Strep A formed a cluster predominantly in this quadrant of the biplot. Antibodies to ScpA, SLO, and SpyAD were positively associated with PC1 and negatively associated with PC2. Those with invasive SDSE formed a cluster predominantly in this quadrant of the biplot. Among the noninvasive cellulitis cohort, ScpA, SLO, and SpyAD were the 3 most common antigens to which there was a positive serological response.

## DISCUSSION

Based on conventional serological criteria using anti-DNB and anti-SLO, 68% of our predominantly older, adult inpatient population with lower limb cellulitis had evidence of BHS infection, consistent with previous research and with our hypothesis [12–14]. When additional vaccine-candidate antigens were incorporated, this number increased to 78%. Those who showed no serological response (22%) may represent a noncellulitis mimic, non-BHS cellulitis, BHS cellulitis with a poor immunological response, or GBS cellulitis. The latter is based on the observation that 3 of 10 participants with proven invasive GBS infection did not respond to any of the tested antigens. As such, BHS may account for even more than 78% of cellulitis cases.

Our finding of 26.8% with a positive anti-DNB and 58.5% with a positive anti-SLO mirrors that found by a large Scandinavian study (30% and 60%, respectively) [14]. GBS does not typically induce an antibody response to SLO and DNB, whereas SDSE often results in a positive anti-SLO but negative anti-DNB [12]. Of the 28 patients with positive anti-DNB and/or anti-SLO serology, 4 were positive for anti-DNB alone and 7 were positive for anti-DNB and anti-SLO, consistent with Strep A infection. The remaining 17 were positive only for anti-SLO, suggestive of either SDSE or Strep A infection. Although there is allelic variation in the deoxyribonuclease B gene and therefore a lack of universal expression in all Strep A isolates [25], prior studies have found this anti–SLO-positive, anti–DNB-negative pattern to be strongly associated with microbiologically confirmed SDSE cellulitis [14], supporting SDSE as the predominant cause of BHS cellulitis.

The dominant role played by SDSE is further supported by the results of the principal component analysis. SDSE responses were characterized by predominant increases in antibodies against SLO, SpyAD, and ScpA. Individually, these 3 antibodies were the most common antigens to which there was a positive serological response among those with cellulitis (58.5%, 46.3%, and 39.0%, respectively). Last, in our study, SDSE was the most common organism recovered from blood cultures among those with cellulitis and bacteremia.

These findings have implications for the potential benefits of a Strep A vaccine, with cellulitis as a target condition. Modelling suggests that a Strep A vaccine meeting the World Health Organization preferred product characteristics could avert 24 million cases of cellulitis across the lifetime of vaccinated individuals [26]. Such estimates are contingent on an accurate understanding of the microbiology of lower limb cellulitis, and improving estimates of Strep A disease burden is a research priority in the World Health Organization Strep A vaccine roadmap [27]. A vaccine with cross-protection against SDSE would further the argument for cellulitis as a vaccine preventable condition, especially if SDSE were responsible for a substantial proportion of cases.

In a historical study, regular dosing of a polyvalent heat and phenol inactivated Strep A vaccine was found to be as equally as effective as monthly benzathine penicillin prophylaxis in preventing cellulitis recurrences, providing proof-of-principle evidence for a potential cellulitis vaccine [28]. Despite these promising early data, immunological correlates of protection conferred by a potential Strep A vaccine remain unknown [29]. If antibodies induced by vaccination can be considered functionally neutralizing, then those that incorporate non-M protein antigens such as SLO, ScpA, and SpyAD may provide protection against not only Strep A cellulitis but also SDSE cellulitis. A study of global SDSE genomes demonstrates that >99% carry genes encoding SLO, ScpA, and SpyAD, with amino acid sequence similarity to Strep A reference sequences >90%, suggesting that Strep A vaccine candidates who incorporate these as antigenic targets may also provide cross-protection against SDSE [30].

In this study, venous insufficiency was the only risk factor that was more common among those with recurrent cellulitis compared to those presenting with a primary episode [7]. Other factors associated with an increased risk for recurrence include tinea pedis, lymphoedema, chronic liver disease, and peripheral vascular disease [5]. Our study may not have been powered to detect these associations and therefore the importance of secondary prevention strategies that address these risk factors cannot be excluded. Those with recurrent cellulitis had higher median GAC and SLO antibody levels at baseline compared to those presenting with a primary episode. Given that GAC antibodies are only rarely induced by group C-variant streptococci strains [16], this may suggest that the preceding episode of cellulitis among those with recurrent infection was caused by Strep A, strengthening the argument that a Strep A vaccine may be a useful approach in cellulitis prevention among this group.

This study has limitations. The lack of a gold standard diagnostic test remains a challenge in cellulitis trials [31]. Although all patients received a clinical assessment by a study member working in the infectious diseases department to confirm a diagnosis of cellulitis, there is small possibility that some patients with a noncellulitis diagnosis may have been inadvertently included. Nevertheless, misdiagnoses would have resulted in an underestimation, rather than overestimation, of the true BHS burden. In contrast, an overestimation may have occurred if patients had experienced a recent noncutaneous BHS infection (eg, pharyngitis). This is felt unlikely given the small proportion of pharyngitis attributable to Strep A among adults compared to children [32]. The higher-than-expected frequency of bacteremia among those with cellulitis may reflect selection bias, given that blood cultures were not systematically collected from all patients and may represent higher severity disease among those in whom cultures were drawn. Alternatively, this may be an overall indicator of higher disease severity among our predominantly older, hospitalized population. It is unclear whether disease severity impacts antibody responses for the analytes assessed in this study and may also be significantly influenced by host and strain-specific factors. Whether the repertoire of antibody responses in invasive infection mirrors that of uncomplicated cellulitis caused by the same organism is also uncertain. Previous studies have demonstrated a lower sensitivity of anti-SLO in impetigo [33, 34], potentially because of cholesterol binding to SLO [35], and could indicate a potential limitation of using a cohort with invasive disease to predict the infecting etiology in those with uncomplicated BHS cellulitis. The lack of widely available, validated assays in clinical use for measuring antibodies to GAC, ScpA, SpyCEP, and SpyAD meant that we could not incorporate an upper limit of normal in our definition of a positive serological response. This was, however, possible for anti-SLO and anti-DNB (200 IU/mL), providing direct comparisons with existing literature.

Future studies should aim to establish an upper limit of normal for the novel vaccine-candidate antigens assessed in this study through large studies on healthy populations with low background Strep A endemicity. Additionally, the immunokinetics of these antibodies are unknown, and thus serial sampling of patients with confirmed invasive BHS infection would be useful to establish the duration of positivity following natural infection and to confirm the optimal time for serological sampling. A comparative analysis of Strep A vaccine candidate antibody immunokinetics between primary and recurrent lower limb cellulitis cases may help determine the relative contribution of the humoral response in preventing infection relapse.

## CONCLUSION

These data indicate that BHS is a common cause of lower limb cellulitis in adults and suggest that SDSE, rather than Strep A, may be the most common pathogen. These findings provide a basis to consider cellulitis as a potential vaccine-preventable condition and suggest that candidate Strep A vaccines that provide cross-protection against SDSE may be useful in secondary prevention. Further characterization of the immunokinetics of antibodies against vaccine-candidate antigens in healthy controls and in those with BHS infection, as well as a greater understanding of the immunological correlates of protection against Strep A and SDSE, will help further inform vaccine development.

## Notes

***Author Contributions.*** M.T.—recruitment of participants, data management, specimen handling, data analysis/interpretation, and manuscript preparation. K.L.—ethics application, recruitment of participants, data management, specimen handling, and manuscript revision. S.H.—recruitment of participants and manuscript revision. C.B.—laboratory analysis and manuscript revision. A.F.—laboratory analysis and manuscript revision. M.M.—laboratory analysis and manuscript revision. E.R.—laboratory analysis, data analysis, and manuscript revision. L.M.—study design and overall supervision of all aspects of this work.

***Financial support*.** This research was supported by an Australian Government Research Training Program (RTP) Scholarship through University of Western Australia.
